# Supplementary material for: Synthesis, Structural Analysis, and Peroxidase-Mimicking Activity of AuPt Branched Nanoparticles
Source: Nanomaterials (Basel). 2024 Jul 8;14(13):1166. doi: 10.3390/nano14131166 (PMC11243270; doi:10.3390/nano14131166)
Supplement: Supplementary file 1 [file nanomaterials-14-01166-s001.zip › nanomaterials-3064490-supplementary.pdf]

Article

Synthesis, Structural Analysis, and Peroxidase-Mimicking Activity of AuPt Branched Nanoparticles

Silvia Nuti [1], Javier Fernández-Lodeiro [1,2,*], Jose M. Palomo [3], José-Luis Capelo-Martinez [1,2], Carlos Lodeiro [1,2] and Adrián Fernández-Lodeiro [1,2,*,†]

[1] BIOSCOPE Research Group, LAQV-REQUIMTE, Chemistry Department, NOVA School of Science and Technology (FCT NOVA), Universidade NOVA de Lisboa, 2829-516 Caparica, Portugal; s.nuti@campus.fct.unl.pt (S.N.); jlcm@fct.unl.pt (J.-L.C.-M.); cle@fct.unl.pt (C.L.)
[2] PROTEOMASS Scientific Society, Praceta Jerónimo Dias, Num. 12, 2A, Sto António de Caparica, 2825-466 Costa de Caparica, Portugal
[3] Instituto de Catalisis y Petroleoquimica (ICP), Consejo Superior de Investigaciones Científicas (CSIC) Marie Curie 2, 28049 Madrid, Spain; josempalomo@icp.csic.es (J.M.P.)
* Correspondence: j.lodeiro@fct.unl.pt (J.F.-L.); fernandez-lodeiro.adrian@ucy.ac.cy (A.F.-L.)
† Current address: Department of Electrical and Computer Engineering, University of Cyprus, Nicosia 2109, Cyprus.

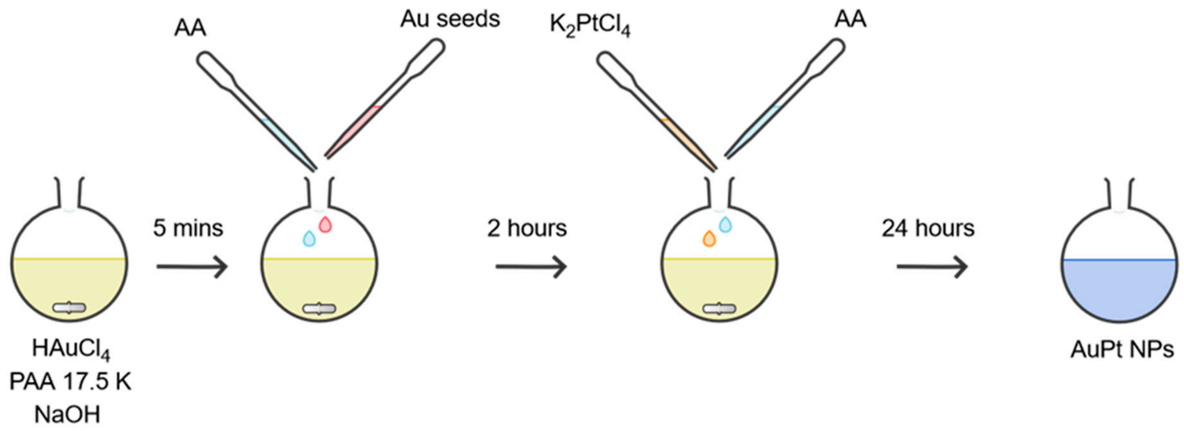

Figure S1. Schematic representation of the AuPt nanostars synthesis.

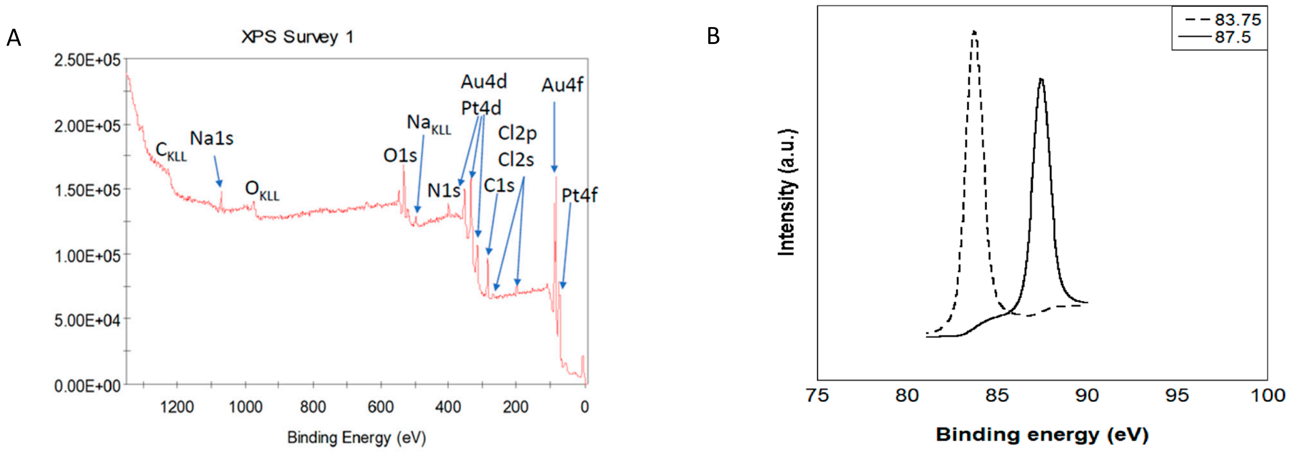

Figure S2. Survey spectra of the sample (left) and enlarged XPS spectra spectrum of AuPt NPs in the Au4f region.

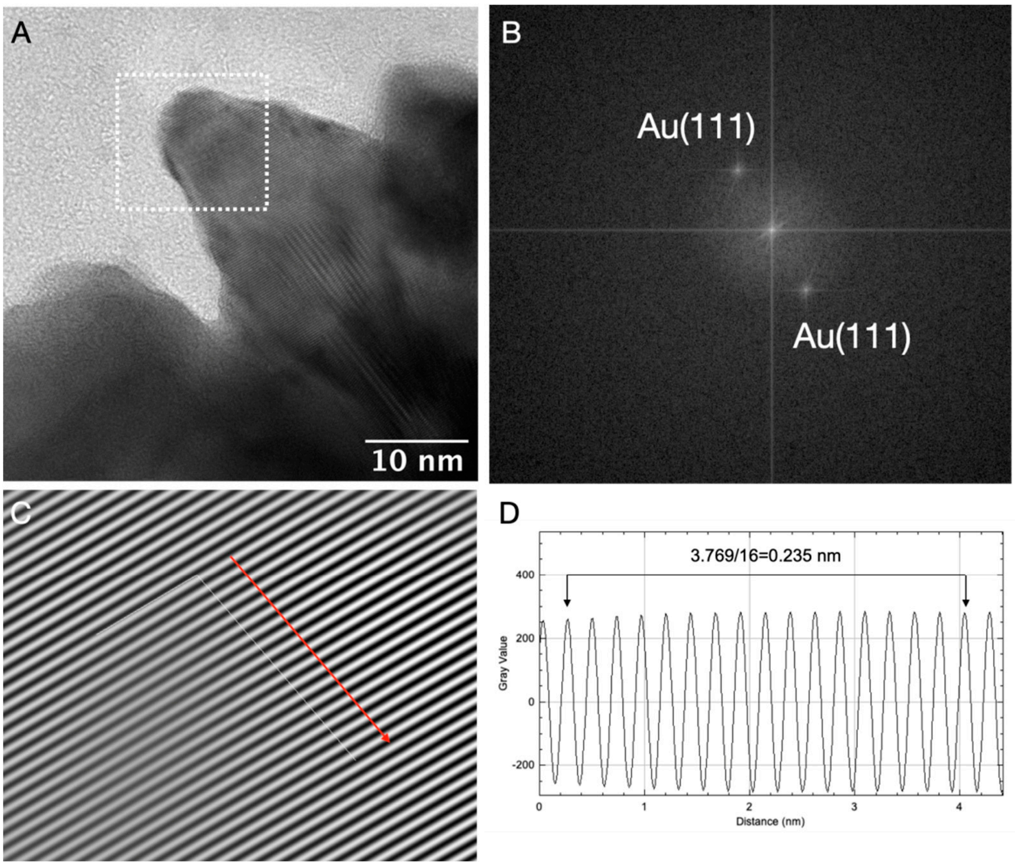

Figure S3. (a) One representative branch in which no apparent metallic Pt deposition occurs. (b) FFT of the white box showed in (a) with bright spots at 0.23 nm. (c) Inverse FFT of one of the bright spot showed in (b). (d) Plot profile corresponding to the red arrow in (c) corresponding to the interplanar distance of 0.235 nm of Au (111).

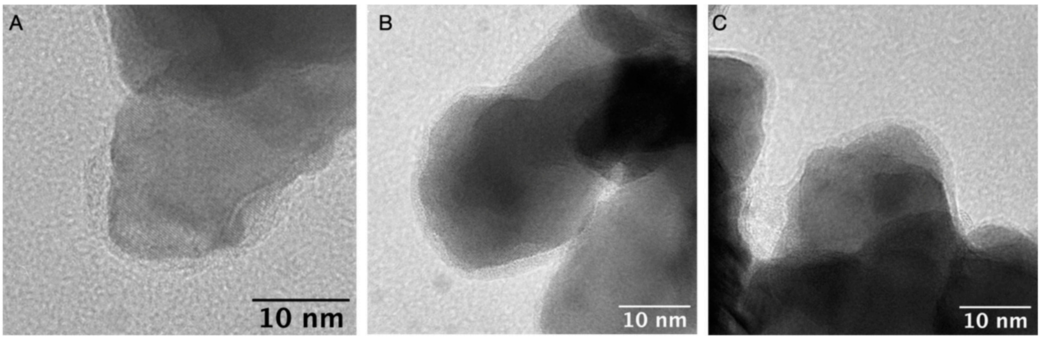

Figure S4. HRTEM of different branched of AuPt NPs showed polycrystalline tips and regions with a ticker non crystalline shell.

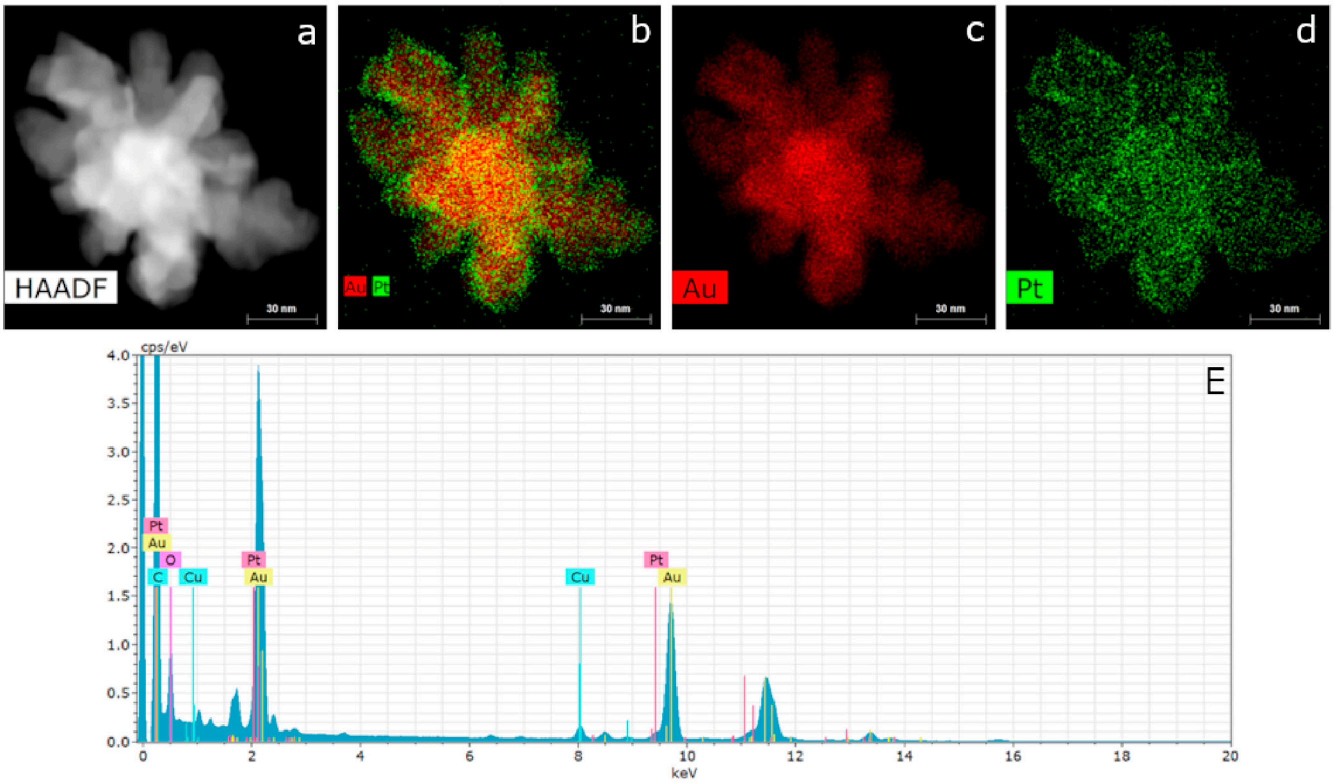

Figure S5. (a) HAADF-STEM image of AuPt NPs. (b) EDX map of AuPt NPs shows Au and Pt distribution. (c) EDX map of AuPt NPs showing Au distribution. (d) EDX map of AuPt NPs showing Pt distribution. (e) EDX spectrum of AuPt NPs showing the presence of Au and Pt.

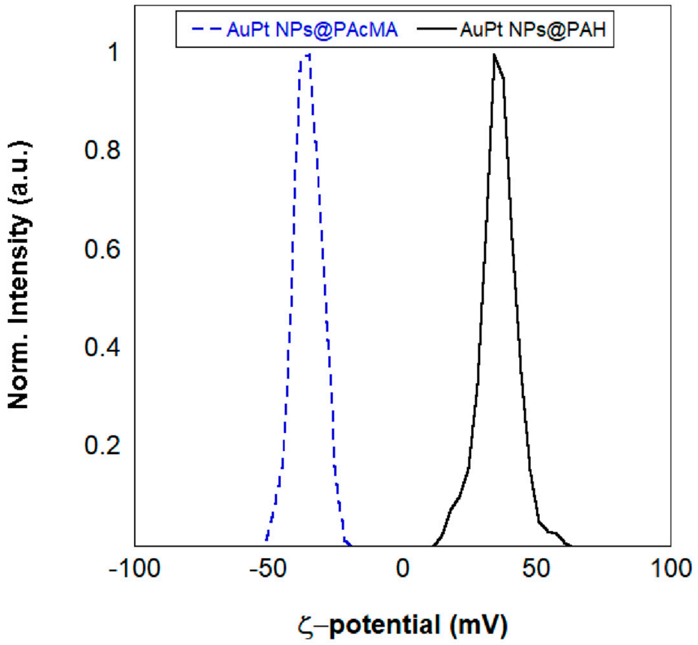

Figure S6. ζ - potential of AuPt and functionalized AuPt@PAcMA NPs.

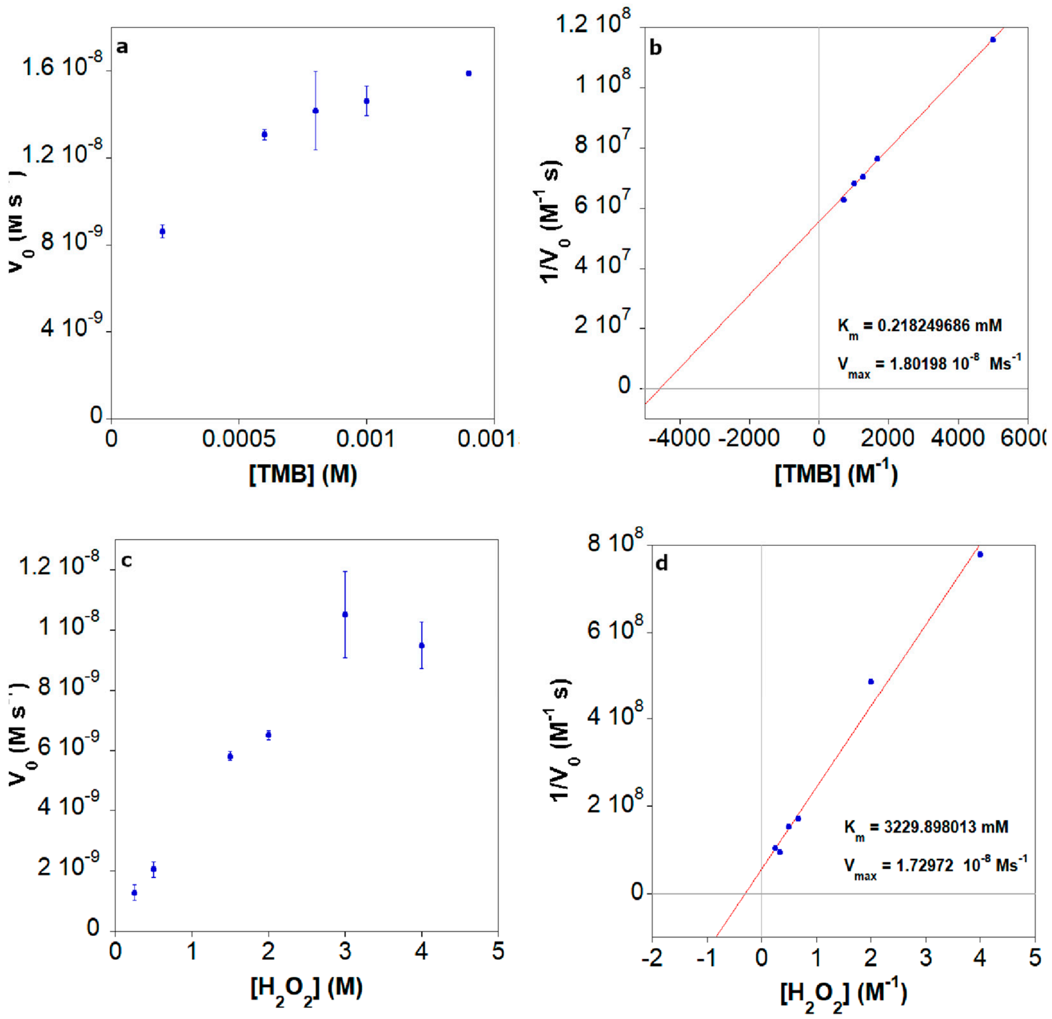

Figure S7. Michaelis-Menten plots for Au NPs nano-enzymatic activity in the oxidation of TMB at (a) constant [H_2O_2] and (c) constant [TMB]. Lineweaver–Burk plots indicating Km and Vmax values at (b) constant [H_2O_2] and (d) constant [TMB]. Error bars represent the standard deviation derived from three independent experiments.

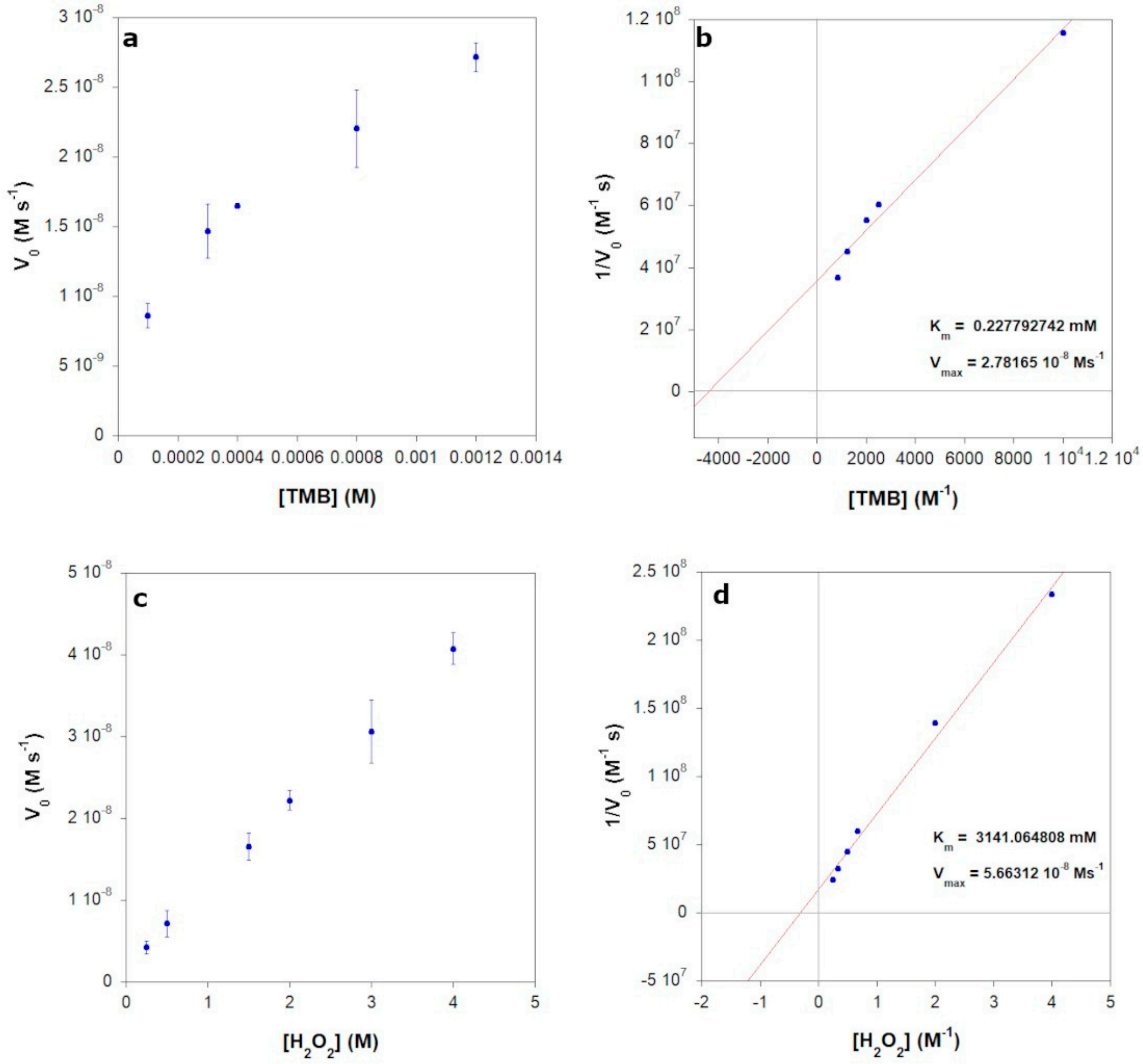

Figure S8. Michaelis-Menten plots for Au@PAcMA NPs nano-enzymatic activity in the oxidation of TMB at (a) constant [H_2O_2] and (c) constant [TMB]. Lineweaver–Burk plots indicating K_m and V_{max} values at (b) constant [H_2O_2] and (d) constant [TMB]. Error bars represent the standard deviation derived from three independent experiments.

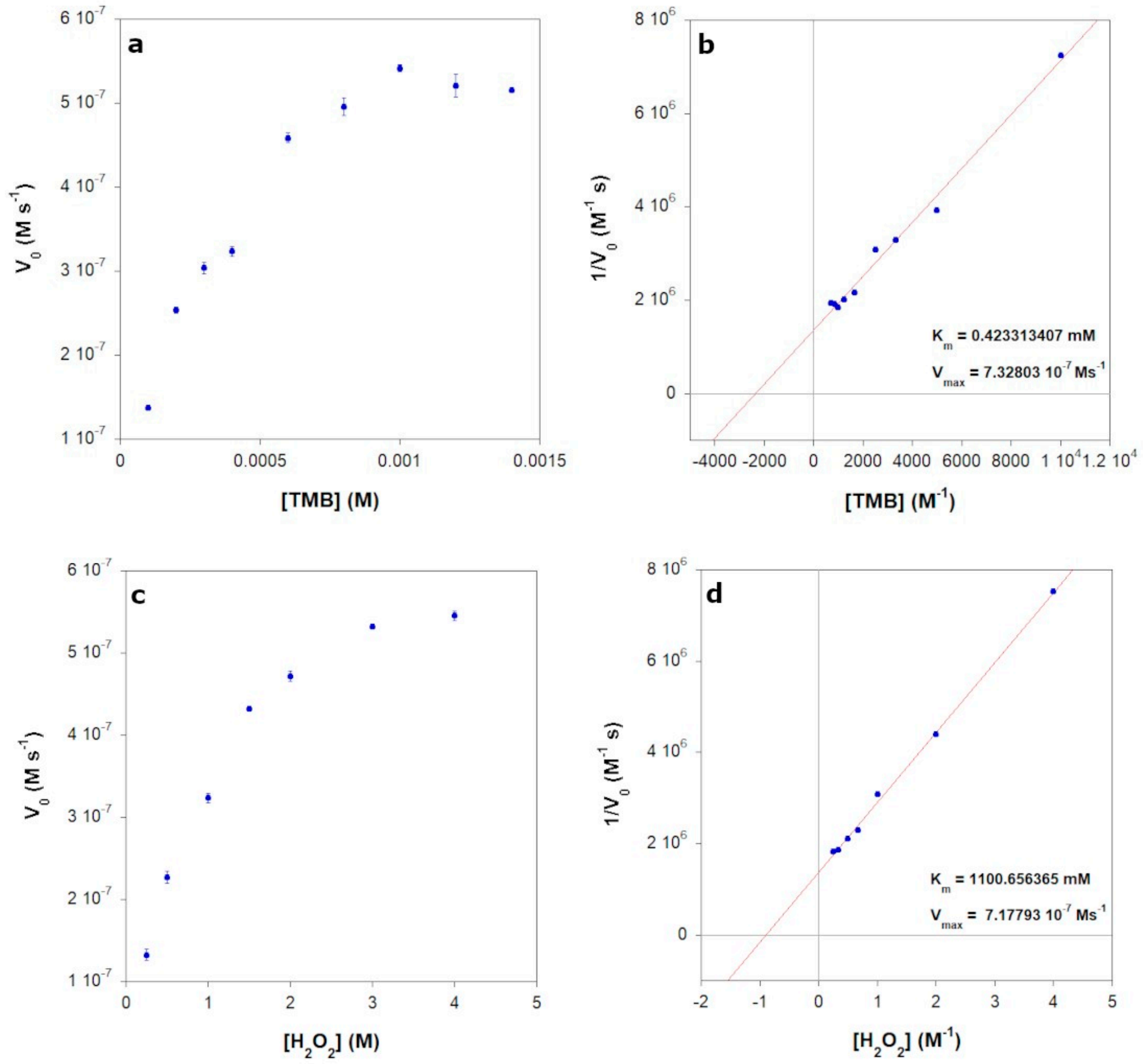

Figure S9. Michaelis-Menten plots for AuPt NPs nano-enzymatic activity in the oxidation of TMB at (a) constant [H_2O_2] and (c) constant [TMB]. Lineweaver–Burk plots indicating Km and Vmax values at (b) constant [H_2O_2] and (d) constant [TMB]. Error bars represent the standard deviation derived from three independent experiments.

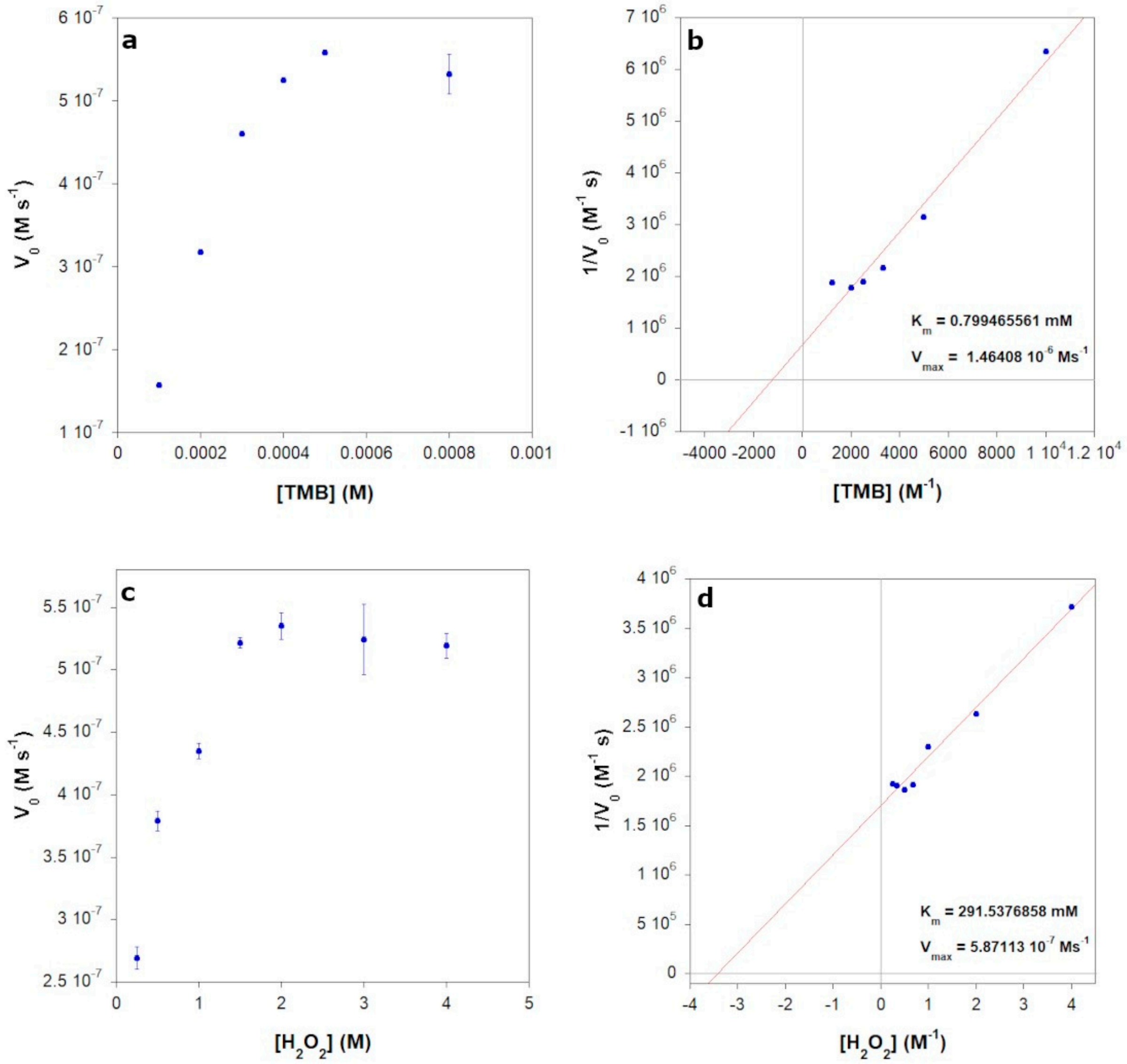

Figure S10. Michaelis-Menten plots for AuPt@PAcMA NPs nano-enzymatic activity in the oxidation of TMB at (a) constant [H_2O_2] and (c) constant [TMB]. Lineweaver–Burk plots indicating Km and Vmax values at (b) constant [H_2O_2] and (d) constant [TMB]. Error bars represent the standard deviation derived from three independent experiments.

Table S1. ICP analysis of AuPt NPs.

	ppm Au	ppm Pt
AuPt	2.268	0.278

Table S2. Comparison of the kinetic parameters (K_m and V_{max}) of the NPs presented in this work with other reported metallic nanozymes in the literature.

Catalyst	*K_m (mM)*		*Vmax (10^{-8} M s^{-1})*		*Ref*
	TMB	*H_2O_2*	*TMB*	*H_2O_2*	
**HRP**	0.434	3.70	10.0	8.71	1
Au NPs	0.21	3229	1.80	1.72	This work
Au@PAcMA NPs	0.22	3141	2.78	5.66	This work
AuPt NPs	0.42	1100.6	73.2	71.7	This work

AuPt@PAcMA NPs	0.80	291.5	146.4	58.7	This work
Au NS	0.155	721	23.7	49	2
Pt NCs	0.63	/	270	/	3
PtC NPs	0.0375	1595.3	14.9	49.3	4
Pt50-PEI	2.02	43.6	115	8.5	5
CA-RhPt NPs	0.129	6.180	68.15	92.7	6
DNA-Pt Nanozymes AG22-Pt1.8	0.0162	117.2	1.93	5.19	7
DNA-Pt AG22-Pt2.1	0.0329	74.4	11.9	30.5	7
DNA-Pt RET2-Pt2.9	0.056	48	58.2	56.8	7
Pt NPs-PVP	2.3	179	281	173	8
Ptn-LP NCs	0.265	2.64	50.3	10.2	9
Ch-Pt NPs	0.018	/	/	/	10
Pt–Ni NPs	0.021	18.36		/	11
SiO_2@Au@Pt	0.417	/	0.021	/	12
Van-Pt_2	1.256	/	66.138	/	13
Au-Pt NCs	0.140	/	21.3	/	14
Citrate Au NPs	0.134	213	9.65	10.6	15
PEG Au NPs	0.155	191	8.32	6.38	15

References:

1. Gao, L.; Zhuang, J.; Nie, L.; Zhang, J.; Zhang, Y.; Gu, N.; Wang, T.; Feng, J.; Yang, D.; Perrett, S.; Yan, X. Intrinsic Peroxidase-like Activity of Ferromagnetic Nanoparticles. *Nat. Nanotechnol.* **2007**, *2* (9), 577–583. https://doi.org/10.1038/nnano.2007.260.
2. Fernández-Lodeiro, C.; Fernández-Lodeiro, J.; Fernández-Lodeiro, A.; Nuti, S.; Lodeiro, C.; LaGrow, A.; Pérez-Juste, I.; Pérez-Juste, J.; Pastoriza-Santos, I. Synthesis of Tuneable Gold Nanostars: The Role of Adenosine Monophosphate. *J. Mater. Chem. C* **2023**, *11* (37), 12626–12636. https://doi.org/10.1039/d3tc01567j.
3. Yu, C.-J.; Chen, T.-H.; Jiang, J.-Y.; Tseng, W.-L. Lysozyme-Directed Synthesis of Platinum Nanoclusters as a Mimic Oxidase. *Nanoscale* **2014**, *6* (16), 9618–9624. https://doi.org/10.1039/C3NR06896J.
4. Bao, Y.-W.; Hua, X.-W.; Ran, H.-H.; Zeng, J.; Wu, F.-G. Metal-Doped Carbon Nanoparticles with Intrinsic Peroxidase-like Activity for Colorimetric Detection of H_2O and Glucose. *J. Mater. Chem. B* **2019**, *7* (2), 296–304. https://doi.org/10.1039/C8TB02404A.
5. Cui, Y.; Lai, X.; Liang, B.; Liang, Y.; Sun, H.; Wang, L. Polyethyleneimine-Stabilized Platinum Nanoparticles as Peroxidase Mimic for Colorimetric Detection of Glucose. *ACS Omega* **2020**, *5* (12), 6800–6808. https://doi.org/10.1021/acsomega.0c00147.
6. Son, S. E.; Gupta, P. K.; Hur, W.; Lee, H. B.; Park, Y.; Park, J.; Kim, S. N.; Seong, G. H. Citric Acid-Functionalized Rhodium–Platinum Nanoparticles as Peroxidase Mimics for Determination of Cholesterol. *ACS Appl. Nano Mater.* **2021**, *4* (8), 8282–8291. https://doi.org/10.1021/acsanm.1c01457.
7. Fu, Y.; Zhao, X.; Zhang, J.; Li, W. DNA-Based Platinum Nanozymes for Peroxidase *J. Phys. Chem. C* **2014**, *118* (31), 18116–18125. https://doi.org/10.1021/jp503242e.
8. Zhang, D. Y.; Liu, H.; Younis, M. R.; Lei, S.; Yang, C.; Lin, J.; Qu, J.; Huang, P. Ultrasmall Platinum Nanozymes as Broad-Spectrum Antioxidants for Theranostic Application in Acute Kidney Injury. *Chem. Eng. J.* **2021**, *409* (September 2020), 127371. https://doi.org/10.1016/j.cej.2020.127371.
9. Fan, L.; Ji, X.; Lin, G.; Liu, K.; Chen, S.; Ma, G.; Xue, W.; Zhang, X.; Wang, L. Green Synthesis of Stable Platinum Nanoclusters with Enhanced Peroxidase-like Activity for Sensitive Detection of Glucose and Glutathione. *Microchem. J.* **2021**, *166* (March), 106202. https://doi.org/10.1016/j.microc.2021.106202.
10. Deng, H.-H.; Lin, X.-L.; Liu, Y.-H.; Li, K.-L.; Zhuang, Q.-Q.; Peng, H.-P.; Liu, A.-L.; Xia, X.H.; Chen, W. Chitosan-Stabilized Platinum Nanoparticles as Effective Oxidase Mimics for Colorimetric Detection of Acid Phosphatase. *Nanoscale* **2017**, *9* (29), 10292–10300. https://doi.org/10.1039/C7NR03399K.
11. Wang, X.; Wei, G.; Liu, W.; Zhang, Y.; Zhu, C.; Sun, Q.; Zhang, M.; Wei, H. Platinum– Nickel Nanoparticles with Enhanced Oxidase-like Activity for Total Antioxidant Capacity *Anal. Chem.* **2023**, *95* (14), 5937–5945. https://doi.org/10.1021/acs.analchem.2c05425.

12. Pham, X.-H.; Tran, V.-K.; Hahm, E.; Kim, Y.-H.; Kim, J.; Kim, W.; Jun, B.-H. Synthesis of Gold-Platinum Core-Shell Nanoparticles Assembled on a Silica Template and Their Peroxidase Nanozyme Properties. *Int. J. Mol. Sci.* **2022**, *23* (12), 6424. https://doi.org/10.3390/ijms23126424.
13. Xue, Y.; Liu, K.; Gao, M.; Zhang, T.; Wang, L.; Cui, Y.; Ji, X.; Ma, G.; Hu, J. Vancomycin Stabilized Platinum Nanoparticles with Oxidase-like Activity for Sensitive Dopamine Detection. *Biomolecules* **2023**, *13* (9), 1–12. https://doi.org/10.3390/biom13091312.
14. Gao, Y. C.; Wang, C.; Zhang, C. X.; Li, H. W.; Wu, Y. Controlled Preparation and Application of Glutathione Capped Gold and Platinum Alloy Nanoclusters with High Peroxidase-like Activity. *J. Mater. Sci. Technol.* **2022**, *109*, 140–146. https://doi.org/10.1016/j.jmst.2021.08.074.
15. Shah, J.; Singh, S. Unveiling the Role of ATP in Amplification of Intrinsic Peroxidase-like of Gold Nanoparticles. *3 Biotech* **2018**, *8* (1), 1–12. https://doi.org/10.1007/s13205-017-1082-1.
